# State cannabis laws and cannabis positivity among fatally injured drivers

**DOI:** 10.1186/s40621-024-00498-1

**Published:** 2024-04-11

**Authors:** Thea Clare Leavitt, Stanford Chihuri, Guohua Li

**Affiliations:** 1 Department of Epidemiology, Columbia University Mailman School of Public Health, 722 West 168th St, New York, NY USA; 2 Department of Anesthesiology, Columbia University College of Physicians and Surgeons, 622 West 168th St, New York, NY USA

**Keywords:** Cannabis, Driving safety, Drug policy, Evaluation research, Laws, Motor vehicle crashes

## Abstract

**Background:**

As of November 8, 2023, 24 states and the District of Columbia have legalized cannabis for both recreational and medical use (RMCL-states), 14 states have legalized cannabis for medical use only (MCL-states) and 12 states have no comprehensive cannabis legislation (NoCL-states). As more states legalize cannabis for recreational use, it is critical to understand the impact of such policies on driving safety.

**Methods:**

Using the 2019 and 2020 Fatality Analysis Reporting System data, we performed multivariable logistic regression modeling to explore the association between state level legalization status and cannabis positivity using toxicological testing data for 14,079 fatally injured drivers. We performed a sensitivity analysis by including multiply imputed toxicological testing data for the 14,876 eligible drivers with missing toxicological testing data.

**Results:**

Overall, 4702 (33.4%) of the 14,079 fatally injured drivers tested positive for cannabis use. The prevalence of cannabis positivity was 30.7% in NoCL-states, 32.8% in MCL-states, and 38.2% in RMCL-states (*p* < 0.001). Compared to drivers fatally injured in NoCL-states, the adjusted odds ratios of testing positive for cannabis were 1.09 (95% confidence interval: 0.99, 1.19) for those fatally injured in MCL-states and 1.54 (95% confidence interval: 1.34, 1.77) for those fatally injured in RMCL-states. Sensitivity analysis yielded similar results.

**Conclusions:**

Over one-third of fatally injured drivers tested positive for cannabis use. Drivers fatally injured in states with laws permitting recreational use of cannabis were significantly more likely to test positive for cannabis use than those in states without such laws. State medical cannabis laws had little impact on the odds of cannabis positivity among fatally injured drivers.

## Background

The use and distribution of cannabis has been prohibited federally in the United States since 1937. However, over the past three decades state-specific cannabis legislation has granted medical and recreational use of cannabis (Pacula and Smart 2017). States with Medical Cannabis Laws (MCL-states) permit the use of cannabis for medical purposes among individuals with qualifying health conditions. In addition, states with Recreational and Medical Cannabis Laws (RMCL-states) also permit the use of cannabis for recreational purposes for individuals aged 21 years and older. States that have No Comprehensive Cannabis Laws (NoCL-states) prohibit and penalize the use of cannabis for any reason (Pacula and Smart 2017). As of November 8, 2023, there are 24 RMCL-states, 14 MCL-states, and 12 NoCL-states [9 states allow the use of low Delta-9-tetrahydrocannabinol (THC), high Cannabidiol (CBD) products for medical reasons in limited situations, and 3 states have not enacted any cannabis laws] (National Conference of State Legislatures 2023). The number of RMCL- and MCL-states, however, are expected to increase, as support for cannabis legalization becomes more pervasive among the US population (Yu et al. 2020).

In general, the prevalence of cannabis use has increased nationally in recent years (Carliner et al. 2017; Yu et al. 2020). It is evident that the prevalence of cannabis use may vary according to the state level legalization status (Martins et al. 2016; Hasin and Aharonovich 2020; Kim et al. 2021; Weinberger et al. 2022). Previous studies have shown that RMCL-states have higher rates of cannabis use and cannabis use disorder among adults, compared to MCL-states and significantly higher rates compared to NoCL-states (Johnson et al. 2012; Carliner et al. 2017; Hasin and Aharonovich 2020). The association between state level legalization status and cannabis use has been partly attributed to the impact of cannabis laws on circumstances surrounding cannabis use (Hopfer 2014; Carliner et al. 2017; Wilson and Rhee 2022). For example, RMCL-states have lower prices, increased product availability and variety, as well as lower perceived risk and social disapproval related to cannabis, all of which may contribute to increased cannabis use (Carliner et al. 2017; Hasin 2018; Hasin et al. 2019; Cerdá et al. 2020).

As the national prevalence of cannabis use has increased over time, cannabis has become the most commonly detected non-alcohol drug among drivers (Brady and Li 2013). The active ingredient in cannabis, delta-9-tetrahydrocannabinol (THC), may impair driving through prolonged response times and reduced vigilance for up to 24 h following consumption (Ronen et al. 2008; Asbridge et al. 2012; Li et al. 2012; Kuypers et al. 2012). One previous study found that when California increased the legalization status of cannabis, transitioning from an MCL-state to a RMCL-state, the prevalence of driving under the influence of cannabis increased (Johnson et al. 2012). Recent studies showed a significant association between RMCL and increased risk of traffic crash injuries and fatalities (Farmer et al. 2022; Adhikari et al. 2023).

Given the national trend towards legalization, the growing prevalence of cannabis use, and the mounting evidence of the dangers associated with cannabis-impaired driving, it is important to better understand the association between state level cannabis laws and cannabis positivity of drivers. Few studies have assessed the association of state level legalization status with the risk of cannabis positivity in fatal motor vehicle crashes based on toxicological testing data. In this study, we tested the hypothesis that drivers fatally injured in RMCL- or in MCL-states are significantly more likely to test positive for cannabis use than those fatally injured in NoCL-states, independent of driver characteristics and crash circumstances.

## Methods

### Data source

Data were taken from the 2019 and 2020 Fatality Analysis Reporting System (FARS). The FARS, sponsored and maintained by the National Highway Traffic Safety Administration, is a census of all crashes that occur on a public road and that results in a fatality to any person within 30 days of the crash in the US (National Center for Statistics and Analysis 2023). Trained analysts extract relevant information relating to the individuals and vehicles involved in, and the circumstances of, the crash. Such data elements include driver characteristics, like sex, age, and race/ethnicity, crash characteristics such as time of day and speed of the vehicle, as well as driver behaviors, like seat belt use and toxicological testing data (National Center for Statistics and Analysis 2023). This study does not meet the definition of human subjects research under 45 CFR 46.

### Study sample and design

During 2019–2020, FARS recorded a total of 169,547 individuals involved in fatal motor vehicle crashes on US public roads. Excluded from the study were individuals who were not drivers (*n* = 64,168), drivers who were not fatally injured (*n* = 57,658), drivers under 15 years of age (*n* = 100), and drivers who died after surviving longer than 1 h following the crash (*n* = 16,792). We only included drivers that died within 1 h of the crash because prolonged survival may lead to false negatives in toxicological testing results or undetectable levels of alcohol and other drugs from metabolism.

**Fig. 1:**
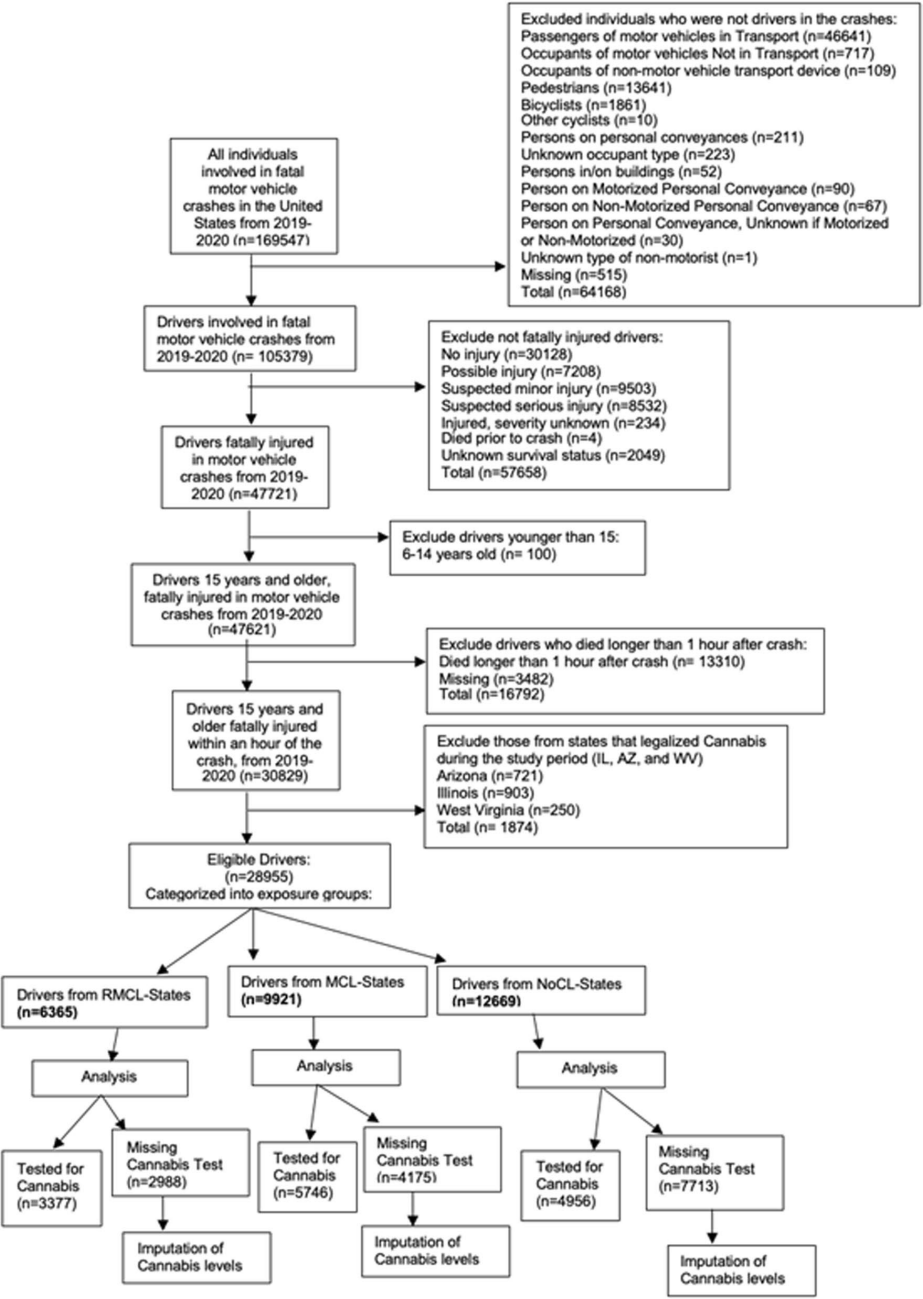
Study population flow diagram, United States, fatality analysis reporting system, 2019–2020

The study sample included drivers from 47 states and the District of Columbia, exclusive of drivers whose crashes occurred in Illinois, Arizona, and West Virginia (*n* = 1874) because their cannabis legalization status changed during the study period (2019–2020) (ProCon.org 2023). Overall, 28,955 drivers were eligible for the study; of them, 14,079 (48.6%) had toxicological testing data available and were thus included in the main multivariable logistic regression (Fig. 1). Toxicological testing data for the remaining 14,876 drivers were not available and were multiply imputed for a sensitivity analysis.

### Study measures

The exposure variable, state level legalization status, refers to the legalization status of cannabis, at the start of the study period (January 2019), in the state that the crash occurred. Drivers included in the study were categorized into three groups: RMCL-states, MCL-states, and NoCL-states. In 2019, 10 US states and the District of Columbia had recreational and medical cannabis laws (RMCLs) (AK, CA, CO, ME, MA, MI, NV, OR, VT, WA, and DC), 20 US states had medical cannabis laws (MCLs) (AR, CT, DE, FL, HI, LA, MD, MN, MO, MT, NH, NJ, NM, NY, ND, OH, OK, PA, RI, and UT), and the remaining 17 states had no comprehensive cannabis legislation (NoCLs).

The outcome variable, cannabis positivity, refers to whether the fatally injured driver tested positive for cannabinoids, based on toxicological testing data recorded in the FARS.

The covariates controlled for in the adjusted model included age, sex, race and ethnicity, restraint use, blood alcohol concentration (BAC), history of driving while intoxicated (DWI), and year of the crash. Driver age was categorized into 5 groups: 15–20, 21–29, 30–39, 40–49, 50–59, and ≥ 60. Race and ethnicity was categorized into 4 groups: Non-Hispanic White, Non-Hispanic Black, Hispanic, and Other. Restraint use was treated as a binary variable, indicating whether or not the driver was using restraint equipment, such as a seatbelt, at the time of the crash. BAC was dichotomized into < 0.08% or ≥ 0.08%, indicating if the BAC was below or above the legal alcohol limit at the time of the crash for all states except UT (Utah is the only state with a BAC legal limit of 0.05%). History of DWI was treated as a binary variable, indicating whether or not the driver had any DWI convictions in the three years prior to the crash.

### Toxicological testing data for cannabinioids

The enhanced FARS data system records all drugs that a driver tests positive for (National Center for Statistics and Analysis 2023). Toxicological testing data are categorized by type: cannabinoids, narcotics, depressants, stimulants, hallucinogens, phencyclidine, inhalants, anabolic steroids, and other drugs (National Center for Statistics and Analysis 2023). The cannabinoid category includes THC products, cannabis, hashish oil, hashish, marinol and other cannabinoids. FARS toxicological testing for the cannabinoid category was primarily based on whole blood specimens (94.6% of the tests), followed by urine samples (3.1%) and other specimens (1.1%) (1.2% of cannabis toxicological testing came from unknown specimen types) to assess the presence of the inactive cannabis metabolite, THC-COOH, to conclude whether the individual tests positive or negative for cannabis use.

### Statistical analysis

Frequencies and percentages of each covariate were computed for fatally injured drivers with toxicological testing data (*n* = 14,079) and those without (*n* = 14,876). Frequencies and percentages of the covariates were further computed for those with toxicological testing data by cannabis positivity status and by state level legalization status. Pearson Chi-Square tests were used to assess the statistical significance in the differences of distributions of these covariates between the groups.

Crude and adjusted odds ratios (aORs) and 95% confidence intervals (CIs) of cannabis positivity associated with state level legalization status were estimated through logistic regression models. The multivariable logistic regression model included the following covariates: age, sex, race, restraint use, BAC, history of DWI, and year of the crash. To assess heterogeneity and the robustness of the associations between state level legislation status and cannabis positivity, stratification analysis was performed by sex, age, year, and BAC.

To address the potential bias due to missing cannabis toxicological testing data, we used the multivariable logistic regression model to impute the missing data for the 14,876 fatally injured drivers with missing/unknown toxicological testing results. Variables included in the multiple imputation model include driver demographic characteristics, crash circumstances, BAC, and DWI history. Our previous study has confirmed that the multiple imputation procedure is a valid approach to handling missing cannabis testing data in the FARS (Chen et al. 2018). Ten imputations were generated and the MIANALYZE procedure was used to summarize estimates of the final model (SAS Institute Inc., Cary, NC). All analyses were performed using SAS 9.4 (SAS Institute Inc., Cary, NC).

## Results

### Driver characteristics

After selection, 6365 (21.9%) of 28,955 drivers were categorized into the RMCL-states, 9921 (34.3%) drivers into the MCL-states, and 12,669 (43.7%) drivers into the NoCL-states. However, only 3377 (23.9%) RMCL-state drivers, 5746 (40.8%) MCL-state drivers and 4956 (35.2%) NoCL-state drivers were included in the main analysis because they had reported toxicological testing data. Compared with drivers included in the analysis (*n* = 14,079), those excluded (*n* = 14,876) due to missing toxicological testing data were similar regarding restraint use (*p* > 0.05), but differed significantly in age, sex, race/ethnicity, BAC, DWI history, as well as time, day, season, and year of crash (*p* < 0.05) (Table 1). Specifically, drivers excluded due to missing toxicological testing data were more likely than those included in the main analysis to be female (22.9% vs. 20.7%, *p* < 0.0001), older (above 30 years) (73.9% vs. 65.2%, *p* < 0.0001), have a BAC above 0.08% (58.5% vs. 41.0%, *p* < 0.0001), and be in a No-CL state (51.8% vs. 35.2%, *p* < 0.0001) (Table 1). Of the drivers with toxicological testing data, 79.3% were male and 59.4% were non-Hispanic White. The mean age of drivers included in the main analysis was 41.8 years.

**Table 1:** Characteristics of fatally injured drivers in motor vehicle crashes in the United States, by those that have cannabis test results and those that do not, fatality analysis reporting system, 2019–2020

Variable	No test results (*N* = 14,876) frequency (column %)	Test results (*N* = 14,079) frequency (column %)	*p* value
Driver demographics			
Age, years			
15–20	932 (6.27)	1317 (9.36)	< 0.0001
21–29	2952 (19.87)	3575 (25.41)	
30–39	2957 (19.90)	2756 (19.59)	
40–49	2337 (15.73)	1919 (13.64)	
50–59	2451 (16.50)	1925 (13.68)	
≥ 60	3229 (21.73)	2577 (18.32)	
Sex			
Male	11,464 (77.10)	11,154 (79.32)	< 0.0001
Female	3405 (22.90)	2908 (20.68)	
Race/ethnicity			
Non-Hispanic White	7907 (61.40)	7770 (59.41)	< 0.0001
Non-Hispanic Black	2092 (16.24)	2302 (17.60)	
Hispanic	2516 (19.54)	2454 (18.76)	
Other	363 (2.82)	552 (4.22)	
Crash circumstances			
Time of day			
Day (7am–6pm)	7717 (51.88)	6929 (49.22)	< 0.0001
Night (7pm–6am)	7159 (48.12)	7150 (50.78)	
Day of the week			
Weekday (Monday–Thursday)	7653 (51.45)	7072 (50.23)	0.0388
Weekend (Friday–Sunday)	7223 (48.55)	7007 (49.77)	
Season			
Fall (September–November)	4150 (27.90)	3634 (25.81)	< 0.0001
Winter (December–February)	3262 (21.93)	2930 (20.81)	
Spring (March–May)	3220 (21.65)	3367 (23.92)	
Summer (June–August)	4244 (28.53)	4148 (29.46)	
Year			
2019	6707 (45.09)	7228 (51.34)	< 0.0001
2020	8169 (54.91)	6851 (48.66)	
Driver behavior			
History of DWI			
No	13,987 (95.50)	13,146 (94.85)	0.0102
Yes	659 (4.50)	714 (5.15)	
Restraint use			
No	8489 (62.19)	8174 (62.42)	0.5507
Yes	5161 (37.81)	4922 (37.58)	
Alcohol level			
< 0.08%	6166 (41.45)	8311 (59.03)	< 0.0001
≥ 0.08%	8710 (58.55)	5768 (40.97)	
State cannabis law			
NoCL	7713 (51.85)	4956 (35.20)	< 0.0001
MCL	4175 (28.07)	5746 (40.81)	
RMCL	2988 (20.09)	3377 (23.99)	

The totals of the variables may vary due to missing values for those variables. Frequency and percentage of missing variables not included

DWI, driving while intoxicated; NoCL-States, states that have no comprehensive cannabis legislation; MCL-States, states with medical cannabis laws; RMCL-States, states with medical and recreational cannabis laws

### Prevalence of cannabis positivity

**Fig. 2:**
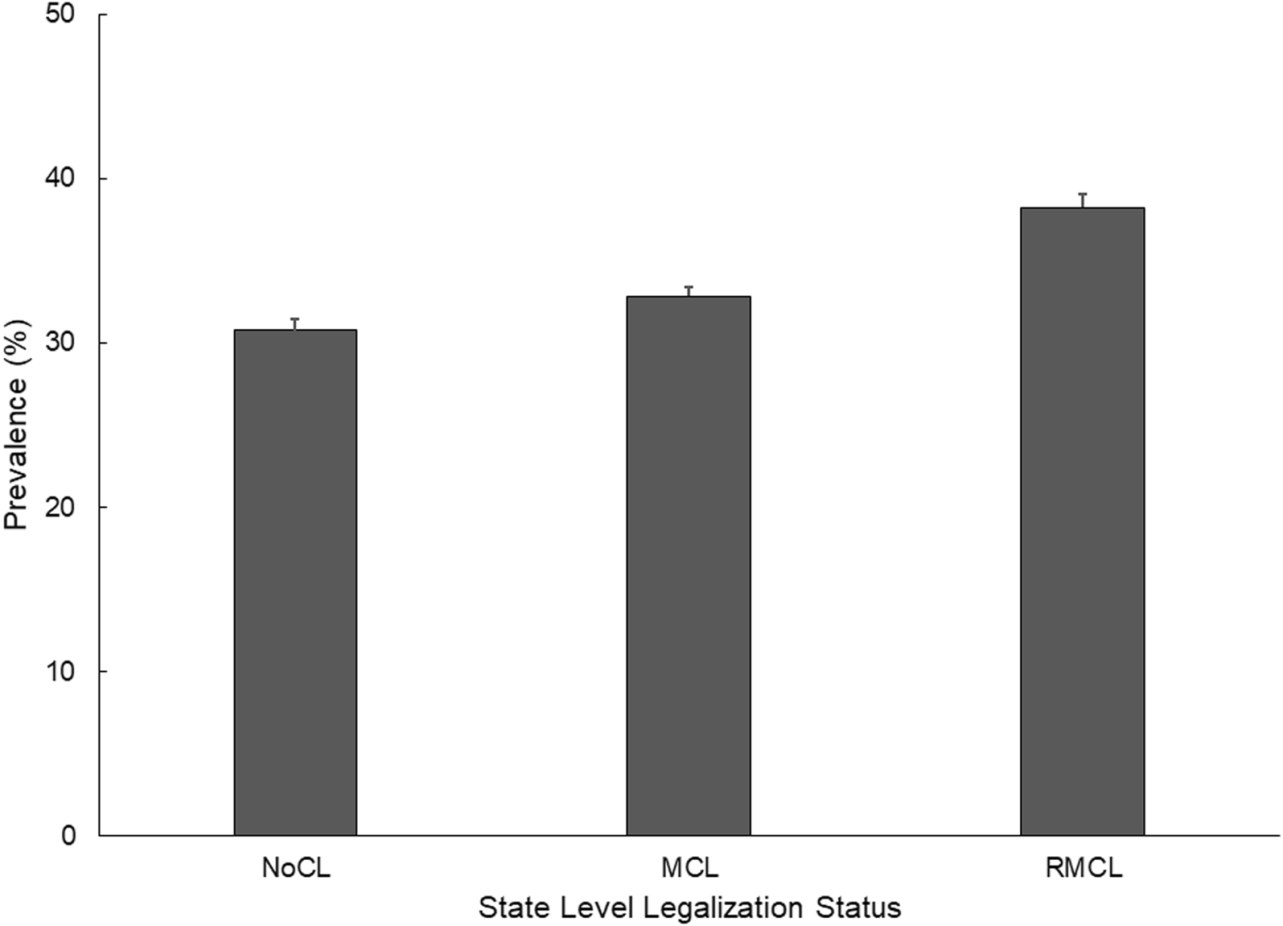
Prevalence and standard error of cannabinoids detected in fatally injured drivers by State Cannabis legalization status, United States, fatality analysis reporting system, 2019–2020. NoCL, states without cannabis legislation; MCL, states with medical cannabis legislation and RMCL, states with recreational and medical cannabis legislation

Overall, 33.4% of the drivers with toxicological testing data were positive for cannabis. Of the 4702 drivers testing positive for cannabis use, 42.5% tested positive for two or more drugs, as categorized by FARS. The prevalence of cannabis positivity was highest among drivers who were fatally injured in RMCL states (38.2%), followed by those in MCL states (32.8%) and those in NoCL states (30.7%) (*p* < 0.001; Fig. 2). Significantly elevated prevalence of cannabis positivity was found among drivers who were under 30 years of age, male, or non-Hispanic black, and those who had a positive history of DWI, were not using restraints, or had BACs ≥ 0.08% (Table 2). Drivers who died in nighttime crashes, crashes occurring in the spring season, or in 2020 also tended to have significantly higher prevalence of cannabis positivity (Table 2).

**Table 2:** Characteristics of fatally injured drivers in motor vehicle crashes in the United States, by cannabis test results, fatality analysis reporting system, 2019–2020

Variable	Total (*N* = 14,079) frequency	Positive cannabis data (*N* = 4702) frequency (Row %)	*p* value
Driver demographic			
Age, years			
15–20	1317	557 (42.29)	< 0.0001
21–29	3575	1606 (44.92)	
30–39	2756	1125 (40.82)	
40–49	1919	621 (32.36)	
50–59	1925	460 (23.90)	
≥ 60	2577	331 (12.84)	
Sex			
Male	11,154	3839 (34.42)	< 0.0001
Female	2908	858 (29.50)	
Race/ethnicity			
Non-Hispanic White	7770	2480 (31.92)	< 0.0001
Non-Hispanic Black	2302	954 (41.44)	
Hispanic	2454	748 (30.48)	
Other	552	177 (32.07)	
Crash circumstances			
Time of day			
Day (7am–6pm)	6929	2065 (29.80)	< 0.0001
Night (7pm–6am)	7150	2637 (36.88)	
Day of the week			
Weekday (Monday–Thursday)	7072	2344 (33.14)	0.5234
Weekend (Friday–Sunday)	7007	2358 (33.65)	
Season			
Fall (September–November)	3634	1212 (33.35)	0.0012
Winter (December–February)	2930	895 (30.55)	
Spring (March–May)	3367	1182 (35.11)	
Summer (June–August)	4148	1413 (34.06)	
Year			
2019	7228	2257 (31.23)	< 0.0001
2020	6851	2445 (35.69)	
Driver behavior			
History of DWI			
No	13,146	4283 (32.58)	< 0.0001
Yes	714	351 (49.16)	
Restraint use			
No	8174	3063 (37.47)	< 0.0001
Yes	4922	1263 (25.66)	
Alcohol level			
< 0.08%	8311	2395 (28.82)	< 0.0001
≥ 0.08%	5768	2307 (40.00)	

The totals of the variables may vary due to missing values for those variables. Frequency and percentage of missing variables not included

DWI, driving while intoxicated

### Multivariate logistic regression analysis

Multivariate logistic regression modeling revealed that the adjusted odds of fatally injured drivers testing positive for cannabis use was 54.0% higher in RMCL-states compared to in NoCL-states (aOR = 1.54, CI: 1.38, 1.72) (Table 3). There was no significant increase in the odds of cannabis positivity when comparing drivers in MCL-states to those in NoCL-states (aOR = 1.09, CI: 0.99, 1.19) (Table 3, Model 1).

**Table 3:** Estimated adjusted odds ratios (aORs) and 95% confidence intervals (CIs) of Cannabis positivity from multivariable logistic regression models, fatality analysis reporting system, 2019–2020

Variable	Model 1 (*n* = 14,079)		Model 2 (*n* = 28,955)	
Driver demographic	aOR	95% CI	aOR	95% CI
Age, years				
15–20	1.25	1.08, 1.46	1.40	1.12, 1.76
21–29	1.23	1.10, 1.38	1.28	1.05, 1.56
30–39	1.00		1.00	
40–49	0.74	0.65, 0.85	0.74	0.59, 0.93
50–59	0.47	0.41, 0.54	0.43	0.36, 0.51
≥ 60	0.25	0.21, 0.29	0.25	0.19, 0.32
Sex				
Male	1.00		1.00	
Female	0.86	0.78, 0.95	0.85	0.73, 1.00
Race/ethnicity				
Non-Hispanic White	1.00		1.00	
Non-Hispanic Black	1.26	1.14, 1.41	1.27	1.16, 1.40
Hispanic	0.67	0.60, 0.75	0.68	0.56, 0.83
Other	0.84	0.69, 1.03	0.88	0.64, 1.20
Crash circumstances				
Year				
2019	1.00		1.00	
2020	1.20	1.10, 1.30	1.45	1.18, 1.79
Driver behaviour				
History of DWI				
No	1.00		1.00	
Yes	1.52	1.28, 1.81	1.66	1.18, 2.32
Restraint use				
Yes	1.00		1.00	
No	1.44	1.32, 1.57	1.47	1.28, 1.69
Alcohol level				
< 0.08%	1.00		1.00	
≥ 0.08%	1.29	1.19, 1.40	1.16	1.04, 1.31
State cannabis law				
NoCL	1.00		1.00	
MCL	1.09	0.99, 1.19	1.05	0.93, 1.20
RMCL	1.54	1.38, 1.72	1.54	1.34, 1.77

Model 1 was based on actual toxicological testing data, and Model 2 was based on multiply imputed data on cannabis testing results

DWI, driving while intoxicated; NoCL-States, states that have no comprehensive cannabis legislation; MCL-States, states with medical cannabis laws; RMCL-States, states with medical and recreational cannabis laws

Regression results further indicated significantly higher odds of cannabis positivity for fatally injured drivers who were male, did not use restraints, had a positive history of DWI, were non-Hispanic Black, had a BAC above the legal limit (0.08%) or crashed in 2020 (Table 3, Model 1).

### Sensitivity analysis

When the multivariable regression model was run by including the multiply imputed cannabis data for the 14,876 fatally injured drivers without toxicological testing data (Table 3, Model 2), the results were generally consistent with the estimates generated from the model based on actual toxicological testing data (Table 3, Model 1).

### Stratification analysis

Results from stratification analysis by driver sex, year of the crash and legal age were generally consistent across strata, indicating that there were no significant interactions (Table 4).

**Table 4:** Estimated Adjusted Odds Ratios (aORs) and 95% Confidence Intervals (CIs) of Cannabis Positivity from Multivariate Logistic Regression, Stratified by Sex, Year and Legal Age, Fatality Analysis Reporting System, 2019–2020

Variable	Sex		Year		Legal Age	
	Female (*n* = 2908) aOR (95% CI)	Male (*n* = 11,154) aOR (95% CI)	2019 (*n* = 7228) aOR (95% CI)	2020 (*n* = 6851) aOR (95% CI)	< 21 Years (*n* = 1317) aOR (95% CI)	≥ 21 Years (*n* = 12,752) aOR (95% CI)
State level legalization status						
No CL	1.00	1.00	1.00	1.00	1.00	1.00
MCL	1.23 (0.99,1.52)	1.06 (0.95,1.17)	1.094 (0.96, 1.25)	1.08 (0.95, 1.23)	1.09 (0.82, 1.46)	1.09 (0.99, 1.20)
RMCL	1.62 (1.25, 2.10)	1.53 (1.36, 1.72)	1.55 (1.34, 1.79)	1.54 (1.31, 1.80)	1.50 (1.07, 2.09)	1.55 (1.39, 1.73)

Adjusted Association, Actual Cannabis Test Data, controlled for age, sex, race/ethnicity, year, previous DWI convictions, restraint use and alcohol level

DWI, driving while intoxicated; NoCL-States, states that have no comprehensive cannabis legislation; MCL-States, states with medical cannabis laws; RMCL-States, states with medical and recreational cannabis laws

## Discussion

The results of this study indicate that state level legalization status is associated with the odds of cannabis positivity detected in drivers fatally injured in motor vehicle crashes. The results show that during 2019 and 2020, fatally injured drivers in RMCL-states were significantly more likely to test positive for cannabis use compared to those in MCL- and NoCL-states. Further, we found no significant difference in the odds of cannabis positivity when comparing fatally injured drivers in MCL-states to those in NoCL-states. These findings are consistent with previous studies that showed RMCL-states are associated with increased rates of driving under the influence of cannabis, when compared to states without recreational legislation (Johnson et al. 2012) and are associated with an increased risk of traffic injuries and fatalities (Farmer et al. 2022; Adhikari et al. 2023).

Our results explored a potential incremental exposure–response relationship between state level legalization status and cannabis positivity; however, no statistically significant incremental exposure–response relationship was found. Instead, we observed a threshold effect of the state level legalization status on the odds of cannabis positivity, occurring at the recreational legalization status level. Our results suggest that it is the recreational legalization of cannabis that is associated with the significantly increased odds of cannabis positivity, and that legalization of cannabis for medical use has little impact on cannabis positivity among fatally injured drivers.

This observed threshold, and the significantly higher odds of cannabis positivity associated with recreational legalization status, may have several explanations. First, the prevalence of cannabis consumption is higher among the general population in RMCL-states, compared to NoCL- and MCL-states, due to increased access and availability (Hopfer 2014; Carliner et al. 2017; Hasin 2018; Hasin et al. 2019; Cerdá et al. 2020; Wilson and Rhee 2022). There is a gap in the literature regarding how disparities in the density of cannabis dispensaries or per capita sales may impact the risk of cannabis use while driving. Future research should explore the relationship between access to cannabis and the risk of cannabis-impaired driving. Second, compared to RMCL-states, where access to cannabis is legal for any individual aged 21 years or older, MCL-states permit the use of cannabis for medical purposes only, among individuals with qualifying health conditions (Bestrashniy and Winters 2015). Because MCL-states have restricted access to cannabis, only accessible for a small fraction of the general population, legalization of cannabis for medical use is unlikely to have a discernible impact on the prevalence of cannabis positivity in the driver population. Third, those granted legal access to cannabis in MCL-states may be less likely to drive in general due to the same health condition that qualifies them for medical cannabis, or they may have received guidance from their prescribing clinician to avoid driving after using cannabis. Finally, residents in RMCL-states may have lower perceived risk and lower social disapproval related to cannabis than those in MCL-and NoCL-states (Carliner et al. 2017; Hasin 2018; Hasin et al. 2019; Cerdá et al. 2020) and are therefore more likely to use it in high-risk situations, such as driving (Johnson et al. 2012; Steigerwald et al. 2020).

### Limitations

Our study has several limitations. First, as with a cross-sectional study design, the observed association between state level legalization status and cannabis positivity does not necessarily infer causality. Second, 51.4% of eligible study subjects were excluded from the main analysis due to missing toxicological testing data, making our results susceptible to selection bias. However, the results of our sensitivity analysis based on multiply imputed toxicological testing data indicate that selection bias is unlikely a significant threat to the validity of our findings. When comparing our results to a 2022 study which obtained toxicology tests from those that were not originally tested, the similarity in the prevalence of fatally injured drivers that tested positive found in both studies (33.4% and 31.7%) indicates that systematic bias within FARS is not a major concern (Chen et al. 2018; Thomas et al. 2022). Third, heterogeneity between the legalization circumstances and regulations of states within each state level legalization status group was not accounted for. Instead of comparing states with different cannabis legalization laws, future studies should compare pre-legalization status to post-legalization status, thus allowing each state to serve as a comparator for itself. Finally, due to the long half-life of THC, our outcome variable (cannabis positivity) does not directly measure cannabis impairment. THC metabolism follows the 3-compartment model of pharmacokinetics (Sempio et al. 2020), and traces of its inactive metabolite, THC-COOH, can be found within the blood specimen up to a week after cannabis consumption whereas adverse effects of cannabis on driving usually disappear after 24 h (Ronen et al. 2008; Richmond et al. 2013). Since 94.6% of FARS toxicological cannabinoid testing data came from whole blood specimens, it is possible that a driver’s positive toxicological testing data for cannabis may be due to an inactive drug metabolite (THC-COOH) from past use, and not necessarily represent current impairment.

## Conclusion

The results of this cross-sectional study provide empirical evidence that state level legalization status is associated with the odds of cannabis positivity in fatally injured drivers. Specifically, we found that drivers fatally injured in states with laws permitting recreational use of cannabis were significantly more likely to test positive for cannabis use than those in states without such laws. State medical cannabis laws had little impact on the odds of cannabis positivity among fatally injured drivers. The findings help further our understanding regarding the public health and safety impacts of state level cannabis legalization. Understanding this relationship between state level legalization status and cannabis positivity is critical for informed policy decisions to ensure that effective interventions are implemented to minimize associated public safety risks and reduce cannabis-related fatal motor vehicle crashes.

## Data Availability

Data for this study came from the Fatality Analysis Reporting System. These data are publicly-available from the National Highway Transportation Safety Administration at https://www.nhtsa.gov/research-data/fatality-analysis-reporting-system-fars.

## Abbreviations

95% CI: 95% Confidence interval
aOR: Adjusted odds ratio
BAC: Blood alcohol concentration
CBD: Cannabidiol
DWI: Driving while intoxicated
FARS: Fatality Analysis Reporting System
MCL-States: States with medical cannabis laws
NoCL-States: States that have no comprehensive cannabis legislation
RMCL-States: States with medical and recreational cannabis laws
THC: Delta-9-tetrahydrocannabinol
